# Colistin Resistance in *Aeromonas* spp.

**DOI:** 10.3390/ijms22115974

**Published:** 2021-06-01

**Authors:** Luis Uriel Gonzalez-Avila, Miguel Angel Loyola-Cruz, Cecilia Hernández-Cortez, Juan Manuel Bello-López, Graciela Castro-Escarpulli

**Affiliations:** 1 Laboratorio de Investigación Clínica y Ambiental, Departamento de Microbiología, Escuela Nacional de Ciencias Biológicas, Instituto Politécnico Nacional, Carpio y Plan de Ayala, Col. Casco de Santo Tomás, Ciudad de México 11340, Mexico; u_gza@hotmail.com (L.U.G.-A.); miguelqbp@gmail.com (M.A.L.-C.); 2 División de Investigación, Hospital Juárez de México, Av Instituto Politécnico Nacional 5160, Magdalena de las Salinas, Gustavo A. Madero, Ciudad de México 07760, Mexico; juanmanuelbello81@hotmail.com; 3 Laboratorio de Bioquímica Microbiana, Departamento de Microbiología, Escuela Nacional de Ciencias Biológicas, Instituto Politécnico Nacional, Carpio y Plan de Ayala, Col. Casco de Santo Tomás, Ciudad de México 11340, Mexico; cecihercor@hotmail.com

**Keywords:** colistin, antimicrobial resistance, *Aeromonas*

## Abstract

The increase in the use of antimicrobials such as colistin for the treatment of infectious diseases has led to the appearance of *Aeromonas* strains resistant to this drug. However, resistance to colistin not only occurs in the clinical area but has also been determined in *Aeromonas* isolates from the environment or animals, which has been determined by the detection of *mcr* genes that confer a resistance mechanism to colistin. The variants *mcr-1*, *mcr-3*, and *mcr-5* have been detected in the genus *Aeromonas* in animal, environmental, and human fluids samples. In this article, an overview of the resistance to colistin in *Aeromonas* is shown, as well as the generalities of this molecule and the recommended methods to determine colistin resistance to be used in some of the genus *Aeromonas*.

## 1. Introduction

Colistin is a lipopeptide antibiotic from the group of polymyxins. It has a cyclic peptide chain that is linked to a fatty acid. Colistin is used in the medical field, since it is an extended-spectrum antimicrobial, and it is used as a last line of treatment in human infections that are caused by Gram-negative bacilli [1]. Until 2016, resistance to colistin was reported in some genera of bacteria intrinsically and contained in the bacterial genophore, until the presence of a gene called *mcr*, present in a plasmid that confers resistance to this antimicrobial, was detected in an *Escherichia coli* strain [2]. After this report, the number of isolates of various origins with *mcr* genes and a colistin resistance phenotype was increased; in addition, it was found that resistance to this molecule could be transferred horizontally [3,4].

The use of colistin as a treatment for infections increased after the appearance of multidrug resistance phenotypes (MDR) in Gram-negative bacilli and the appearance of carbapenemase-producing enterobacteria type KPC (*Klebsiella pneumoniae* carbapenemase) or NDM (New Delhi metallo-β-lactamase), in addition to Gram-negative bacilli classified as XDR (extensively drug resistant) that continue to appear, especially in bacteria such as *Klebsiella pneumoniae*, *Pseudomonas aeruginosa*, and other Gram-negative bacilli, such as *Aeromonas*, which is also of medical and veterinary importance and is isolated from environmental samples [5,6].

*Aeromonas* is a Gram-negative bacillus, positive for oxidase and catalase tests, a glucose fermenter, and it is resistant to vibriostatic O/129 (2,4-diamino-6,7-diisopropylpteridine) [7]. In humans, it can cause intestinal and extra-intestinal infections. It is important in the medical area, mainly in patients with diarrhea, or with infections in the skin and soft tissue; moreover, it can cause bacteremia, which progresses to sepsis, or endocarditis [8,9].

The genus *Aeromonas* is widely distributed in diverse ecosystems; however, it is a bacterium native to aquatic systems, hence the largest number of isolates are from water. Isolates have been obtained from drinking water, wastewater, bottled water, seawater, and deep and surface water samples. Food isolates have been obtained from vegetables, fruits, pork, poultry, and beef, as well as seafood and fish. In animals, it is considered a pathogen, especially in fish, in which it can cause furunculosis, ulcers, and hemorrhages, among other diseases. This pathogen has also been isolated from infections in rabbits, dogs, cats, chickens, horses, and crustaceans [8,9].

## 2. The Colistin Situation

**Figure 1:**
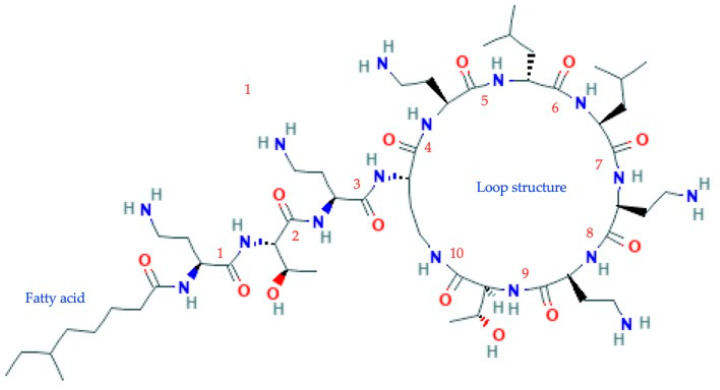
Polymyxin or colistin structure. Basic structure of polymyxin E composed of a chain of 6-methyl-octanoic acid (fatty acid), linked to a cyclic lipopeptide of 10 amino acids. 1, diaminobutyric acid (L-Dab); 2, threonine (L-Thr); 3, diaminobutyric acid (L-Dab); 4–5, diaminobutyric acid (L-Dab); 6, leucine (D-Leu); 7, leucine (L-Leu); 8–9, diaminobutyric acid (L-Dab); 10, threonine (L-Thr). The residues in position 6 of D-Leu and L-Leu in position 7 determine polymyxin E. Amino acid substitution at positions 6 and 7 determines the colistin subtype. The loop is formed by the amino acids from position 4 to 10. This cyclic peptide is composed of non-proteinogenic amino acids (D-Leu; L-Dab) and contains an isopeptide bond linking amino acids 4 and 10. These characteristics make the peptide resistant to the action of most peptidases/proteases and indicate that the compound originates from a non-ribosomal protein synthesis (NRPS) biosynthetic pathway. Modified from PubChem (https://pubchem.ncbi.nlm.nih.gov/compound/Colistin#section=Names-and-Identifiers, access date: 21 May 2021).

Colistin is a lipopeptide molecule that is included in the polymyxin family. This molecule has a peptide chain that is made up of 10 amino acids and is linked to a fatty acid. It has surface-active properties that alter the permeability of the Gram-negative membrane in contact with lipopolysaccharides (LPS), resulting in cell death due to the electrostatic attraction that is exerted between the positive charge of the amino groups and the anions, phosphate, and carboxylate (Figure 1) [1,3,4].

This molecule contains a structure called a cyclic loop, formed by the seven amino acids that are among the amino group of the diaminobutyric acid side chain in position 4 and the carboxyl group of the terminal carbon of residue number 10 of threonine (Thr). This loop is linked by a chain of three amino acids to the amino terminal fatty acid [1].

Colistin’s mechanism of action is focused on the membrane structure of Gram-negative bacteria; they have the outer membrane that is a barrier of the passage of various substances, including antimicrobials. This membrane has a phospholipid layer inside and LPS on the outside. The LPS structure has three domains: lipid A, which is the domain that is anchored to the membrane and the hydrophobic and endotoxic portions of the structure; the O antigen, an oligosaccharide polymer; and the nucleus or core, which is also an oligosaccharide, and is the domain that connects lipid A to antigen O and is divided into the inner core and the outer core [1,3,10,11,12].

**Figure 2:**
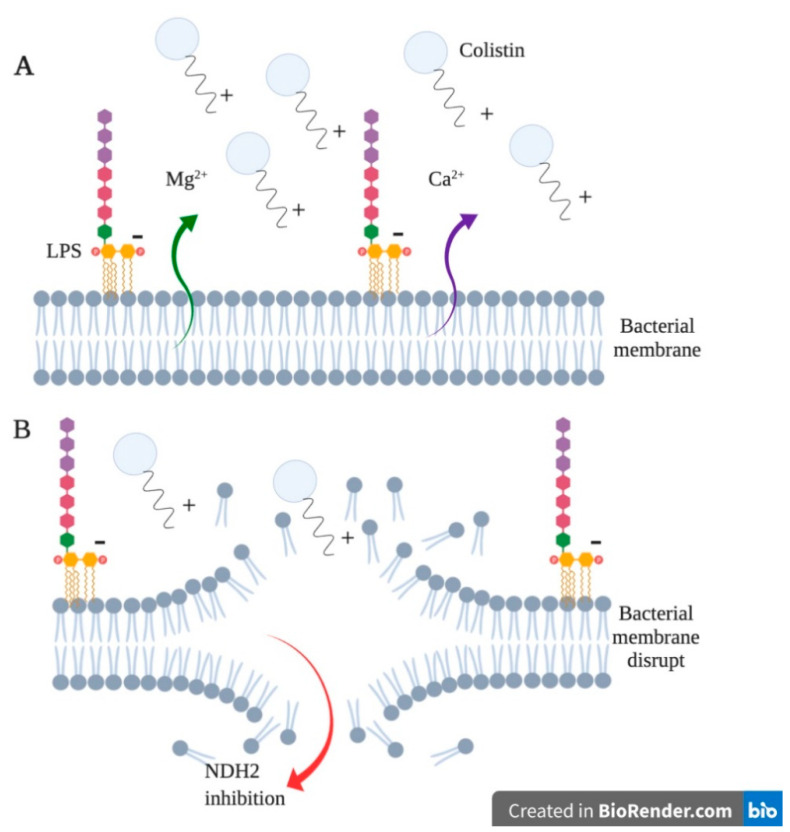
Colistin mechanism of action. (**A**) The positively charged colistin molecule interacts with the negative charges of the LPS membrane, resulting in the release of Ca^2+^/Mg^2+^ ions. (**B**) This interaction results in a membrane disruption, therefore, the output of Ca^2+^/Mg^2+^ ions is generated. Finally, colistin enters the cytoplasm and inhibits the activity of the oxidoreductases enzyme (NDH_2_). Generated from this work.

Lipid A is a glucosamine disaccharide linked by a β-1,6 bond, esterified at four positions with saturated fatty acids, and phosphorylated at ends 1 and 4. Two divalent cations participate in the mechanism of action of colistin, Mg^2+^ and Ca^2+^, associated with the phosphoesters of lipid A. These cations keep the adjacent LPS molecules together and stable, in such a way that the membrane of the Gram-negative bacterium exerts a dual function, as a mechanical barrier and at the same time an electrostatic barrier with a high repulsive anionic charge that is generated by the phosphoesters of lipid A, and by the phosphates and carboxylates of the core sugars and the O antigen (Figure 2) [1,3,10,11,12].

### 2.1. Colistin Activity Spectrum

Colistin resistance rates are generally low, in contrast to carbapenems, which are constantly increasing. The limited therapeutic options against MDR microorganisms such as *A. baumanni* or *P. aeruginosa* lead to an increased use of colistin in the treatment of this type of microorganism. For this reason, it is controlled in the hospital area and the use of other antimicrobials or the combination of two is preferred, before resorting to colistin [13].

The use of colistin is focused mainly on the family *Enterobacteriaceae* and non-fermenting bacilli. Within these genera, there are groups with intrinsic resistance to colistin, such as *Neisseria* spp., *Stenotrophomonas maltophilia*, *Brucella* spp., *Proteus* spp., *Providencia* spp., *Serratia* spp., and *Burkholderia* spp., which are outside the spectrum of this antimicrobial, and likewise, Gram-positive cocci and strict anaerobes, such as *Bacteroides fragilis* (Table 1) [3].

In addition to the bacterial groups of medical importance with colistin resistance that have been isolated from clinical samples and other environmental sources, from animals or from food, there are other Gram-negative bacilli that are important, such as human and animal pathogens, which have been isolated from various sources, such as the genus *Aeromonas*, from which their resistance to this drug has been determined and their probable responsibility that *mcr* genes are resistant to it. Resistance to colistin is also present in isolates from environmental samples, from which antimicrobial resistance has been documented [14,15,16,17,18].

### 2.2. Colistin, Pharmacology and Application

Currently, the use of colistin has increased in combination with other drugs, such as phosphomycin and tigecycline, as has the emergence of resistance to carbapenems, in combination therapy, double or triple, due to the shortage of active pharmacological molecules. This was derived from the presentation of resistant phenotypes of KPC or NDM, and those Gram-negative rods classified as XDR that were increased to include Gram-negative rods and non-fermenter bacilli [1,2,3].

Among the drugs that are part of the treatment strategy against MDR microorganisms are polymyxins B and E (colistin). Although these antibiotics have adverse effects, such as nephrotoxicity and neurotoxicity, they are one of the last alternatives available for human infections caused by multidrug-resistant Gram-negative bacilli. In contrast, genes such as *mcr* have been found, whose products are related to colistin resistance [11,12].

## 3. Antimicrobial Resistance in *Aeromonas*

The molecular basis of antimicrobial resistance in *Aeromonas* spp. has been widely studied, but their importance in the hospital area as a cause of outbreaks is not fully established. Likewise, reports on resistance are varied regarding the origin of isolation and the type of antimicrobials tested in vitro [7]. The *Aeromonas* resistance profile has not changed significantly; until now, the mechanism of action of inducible chromosomal β-lactamases and carbapenemase expression has been suggested as being the main resistance mechanism for *Aeromonas* species. Three classes of β-lactamases are recognized in *Aeromonas*; one of class C cephalosporinase, one of class D penicillinase, and one of class B metallo-β-lactamase (MBL) [9,19].

The occurrence of MDR-type *Aeromonas* spp. isolates has been increasing. Different authors have suggested that antimicrobial resistance in the clinical setting is closely related to resistance mechanisms detected in environmental isolates [20]. In the genus *Aeromonas*, the occurrence of MDR strains is equivalent, due to their origin in aquatic environments, which is attributed to the extensive use of antibiotics in aquaculture. Therefore, this environment becomes an ideal setting for the acquisition of these mechanisms of resistance to antimicrobials and other toxic agents [21].

### Colistin Resistance in Aeromonas

Since the report in 2016, where it was shown that colistin resistance can be encoded by the *mcr* genes detected within a plasmid, it was determined that these genes are not only in bacterial genophores but can also be present in mobile genetic elements as plasmids [2]. From this report, attention was paid to the search for and detection of these genes in different bacterial genera, mainly those of medical importance, but also isolated from other sources, such as the environment or animals. In *K. pneumoniae*, *P. aeruginosa*, or others, such as *Aeromonas* genus, *mcr* gene variants have been detected, and the reports are increasing [22,23].

Colistin resistance in *Aeromonas* has been reported in several regions of the world, mainly in Europe and Asia. Resistance to this antibiotic has been reported in Latin America in other genera, but not in *Aeromonas*. The detection of colistin resistance is more common in the clinical area; however, colistin-resistant strains of *Aeromonas* have been isolated from other origins that have been detected, from which investigations and reports have emerged in the world. The extensive use of antibiotics in aquaculture and in human treatment has led to an increase in the resistance of this genus to antimicrobial drugs [20,21].

The species *A. dhakensis*, *A. hydrophila*, *A. caviae*, and *A. veronii* are considered the main causes of human infections that can cause infection in wounds, diarrheal syndromes, and other clinical presentations [9]. Commonly, the isolates do not present resistance to antimicrobials; however, MDR isolates have still appeared, and in recent years the report of *Aeromonas* isolates from clinical samples and from various sources with resistance to colistin has increased [24]. This resistance has been investigated in *Aeromonas* spp. isolates, by means of disk diffusion test and by minimum inhibitory concentration (MIC), showing the MIC method to be more effective. Induction of colistin resistance in the strains showed an 85% increase after overnight incubation in a tube with *Müller*–*Hinton* broth and a 50 µL colistin disk. This result allowed the establishment of a phenotypic marker in the *Aeromonas* isolates [25].

In an *Aeromonas veronii* isolate from chicken meat, two adjacent genes with colistin resistance markers, called *mcr-3.3* and *mcr-3*-like, were detected in the genophore. The result had 95.2 and 84.19% identity in the nucleotide sequence, when compared to the *mcr-3* gene of an *E. coli* of porcine origin [26].

The evidence of the *mcr* genes in *Aeromonas* was demonstrated by a group of scientists who analyzed a total of 6497 strains that were collected in 13 provinces of China between 2016 and 2017. In these samples, the presence of the *mcr-3* genes was detected by PCR. The *mcr-3* gene was detected in 49 strains only, of which eight strains corresponded to the genus *Aeromonas*, two *A. hydrophila* strains, one with *mcr-3.8* variant, and one with *mcr-3.9* variant, one *A. caviae* with *mcr-3.1* variant, and one *A. media* with *mcr-3.6* variant. Of the four remaining strains, one each were of *A. veronii*, *A. media*, and *A. caviae*, and one was *Aeromonas* spp. with *mcr-3* without variant. All the strains were grouped into a subclade, after the phylogenetic analysis of the sequences of the *mcr-3* genes detected in the strains [18].

Another group of researchers found four *Aeromonas* isolates with the presence of the *mcr-3* gene through PCR, while the *mcr-1* or *mcr-2* genes were not detected. Each of the four isolates with *mcr-3* genes presented a different variant each; these presented identities in the amino acid chain were of 95 to 98% compared to the original protein MCR-3. These variants of the protein were designated as MCR-3.6 obtained from the *A. allosaccharophila* strain isolated from *Leuciscus idus*, MCR-3.7 for the protein detected in the *A. media* strain isolated from *Meleagris gallopavo*, MCR-3.8 for that detected in the *A. jandaei* strain isolated from a *Cyprinus carpio* carp, and MCR-3.9 for the protein of the *A. hydrophila* strain of *Cyprinus carpio*. The isolate with the *mcr-3.9* gene also contained an additional *mcr-3.8* gene in the MIC test, with colistin showing an MIC ≥ 128 mg/L higher compared to the other isolates [27].

The reports include a new variant of the *mcr-3* gene in *A. caviae*, also detected in *Proteus mirabilis* and *E. coli* that were isolated from a domestic duck. These strains were obtained from sewage samples from free-range ducks, which were raised near a river in the suburban area of Qingdao, Shangdon Province, in China. The presence of the *mcr-3* gene was demonstrated in 1 of 15 samples processed in this study. The result was obtained by detection of the *mcr* gene directly in the sample; the positive sample was seeded in a CHROMagar plate from Biomerieux^®^, France, to which 2 mg/L of colistin was added. Based on the above, three positive strains were detected for *mcr-3* gene. *A. caviae* 17AC, *P. mirabilis* 17PM, and *E. coli* 17EC strains were identified by MALDI-TOF (Matrix-Assisted Laser Desorption/Ionization-Time of Flight) technology, and by *16S rRNA* gene sequencing [27]. In another study, the prevalence was determined, complemented by a genetic analysis of the *mcr-3* gene in *Aeromonas* species. These isolates were obtained from human rectal exudates, meat for human consumption, and environmental water samples [28].

The variant *mcr-5* gene of colistin resistance was detected in an *A. hydrophila* strain isolated from a fecal sample from a backyard pig. In this case, the *mcr-5* gene was detected in a plasmid with 7915 base pairs (bp) named pI064-2. Additionally, they analyzed the possibility of transforming the *A. hydrophila* strains susceptible to colistin into a resistant strain [29].

Various mechanisms of resistance to colistin, in addition to the mechanism mediated by *mcr* genes, have been described in some bacteria, including *P. aeruginosa*, *A. baumannii*, members of the *Enterobacteriaceae* family, such as *E. coli*, *Salmonella* spp., and *K. pneumoniae*—they have an acquired resistance against colistin. However, the possibility of the appearance of strains resistant to this antibiotic should be monitored, due to the presence of mutations, new mechanisms, or adaptations [11]. 

**Figure 3:**
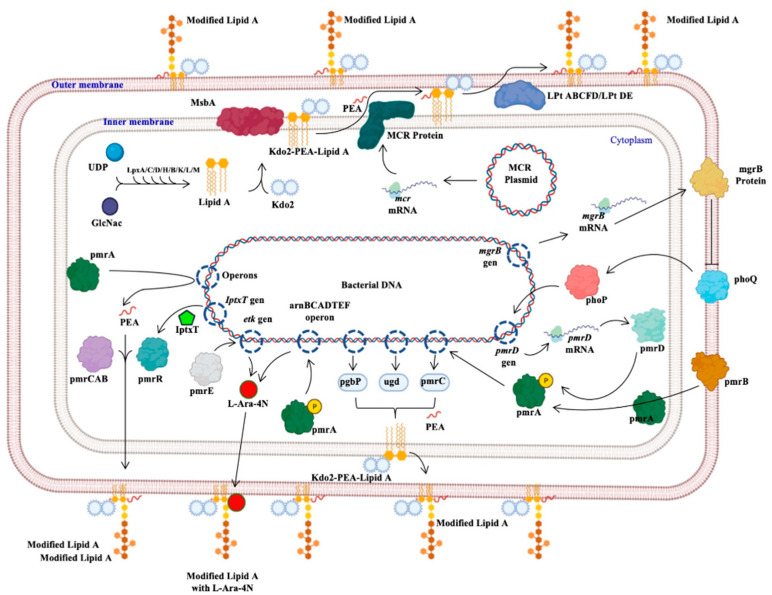
Bacterial mechanisms of resistance to colistin. The different mechanisms of resistance to colistin described in Gram-negative bacilli and other bacteria are presented: the mechanism mediated by the *mcr* genes, those regulated by the phoP and phoQ proteins, others regulated by the pmr proteins or by the *iptxT* and *etk* genes, and operons that encode activation proteins of pmr proteins [11,30,31,32]. Generated from this work.

The protein generated by the *mcr* genes confers resistance to polymyxins; this protein, called MCR, from the inner membrane, adds a molecule of phosphoethanolamine (PEA) to lipid A of lipopolysaccharide (Kdo2-PEA-Lipid A), synthesized by the binding of uridine diphosphate (UDP) and N-acetyl glucosamine (GlcNac) mediated by Lpx proteins (C, D, H, B, K, L, M). The product binds to 2-keto deoxioctanate acid (Kdo2), which is transported by the MsbA protein to the inner membrane, where the PEA molecule is added. The Lpt protein complex (ABCFD/DE) transports the modified LPS that generates resistance to colistin, since the negative charge with which it interacts was modified (Figure 3) [30,31,32]. 

Another mechanism of resistance to colistin is the two-component system composed of the membrane proteins phoP and phoQ. This mechanism is regulated by the expression of the *mgrB* gene, and when the phoQ protein is activated, it acts on phoP, that functions as an inducer of the *prmD* gene, which phosphorylates the prmA protein; additionally, the prmA protein can be activated by the prmB protein. The phosphorylated prmA protein activates the expression of the *pgbP*, *ugd*, and *prmC* genes, which independently add a PEA molecule to the LPS, generating resistance to colistin in the bacteria. Likewise, the prmA protein can induce the expression of operons, whose composition has not been determined, and which adds a PEA molecule mediated by the prmC, A, and B proteins. Furthermore, the lptx protein, with the help of the prmR protein, can also add a PEA to the LPS (Figure 3) [11,30]. 

The last colistin resistance mechanism described in this article is the one that adds a 4-amino-4-deoxy-L-arabinose (L-Ara-4N) to the LPS, in which the prmA protein induces the expression of the arnBCADTEF operon; additionally, the prmE protein induces the expression of the *etk* gene, which is also capable of adding an L-Ara-4N to the LPS. This modification generates a different structure in the LPS that prevents colistin from interacting with Lipid A of LPS; therefore, colistin is unable to perform its function (Figure 3) [11,30,31,32].

Until now, only the *mcr-1*, *mcr-3*, and *mcr-5* gene variants have been reported in the genus *Aeromonas*. The variability in the presence of these genes in Gram-negative bacilli may be due to the diversity of their origin, the plasticity of the genus to receive external genetic material, or the selective pressure exerted by both the environment and exposure to antimicrobials on the health environment [33].

## 4. Detection Methods of Colistin Resistance

In the 2021 version of the CLSI, methods for the determination of resistance or susceptibility to colistin by MIC are described. The accepted methods are microdilution in broth, elution in broth, and diffusion in agar. These methods are indicated for the *Enterobacteriaceae* family and for *P. aeruginosa*; however, there are no indications for other Gram-negative bacilli, such as *Aeromonas* [34].

### 4.1. Elution Method in Broth

The CLSI 2021 (M-100) indicates the use of four tubes with 10 mL of Müeller–Hinton broth with cation adjustment for Ca^2+^ and Mg^2+^ in each one. Aseptically, one disc of colistin (10 μg/mL) to tube “1”, two discs to tube “2”, and four to tube “4” are added; the control tube will not have a disc. The tubes are completely closed after adding the colistin discs and left to stand for no more than an hour, so that the colistin elutes from the discs in the broth, at room temperature. These tubes are considered as the stock or stock solution for use with each strain [6,22,35].

From growth of 24 h in a plate of the strains to be tested, an inoculum will be adjusted with sterile physiological solution. From the adjusted inoculum, 50 μL are placed in each of the tubes marked “1”, “2”, “4”, and the control, to reach a final concentration of approximately 7.5 × 10^5^ CFU/mL. Additionally, it is recommended to inoculate blood agar plates with 5% sheep blood, to check viability and purity. The inoculum is mixed with each tube with colistin softly, so that the colistin does not adhere to the walls of the tube or the cap. The tubes are incubated between 33 and 35 °C for 16–20 h, taking time to observe the test [6,36].

For results interpretation, bacterial growth is expected in the control tube to validate the test. The MIC will be that of the tube with no growth and with the lowest concentration of colistin. If the MIC is ≤2 μg/mL it is intermediate, or resistant if the MIC is ≥4 μg/mL. If inconsistency is shown in the results, for example, bacterial growth in tube 1 and no bacterial growth in tube 2, the method is repeated. These results may be due to the presence of contamination in the inoculum, presence of heteroresistance, errors in tube inoculation, or inappropriate concentrations in elution [6,36,37].

### 4.2. Plate Test Method

Initially, the strain purity is analyzed in a plate incubated for 24 h on plates with colistin agar, and the concentration is adjusted to 0, 1, 2, and 4 μg/mL. The tube of the McFarland Nephelometer scale is adjusted to 0.5 in 4–5 mL of sterile saline solution, then diluted 1:10. Up to 10 strains of the dilution are seeded on colistin agar plates with a bacteriological loop adjusted to 10 μL. Then, they are incubated between 33 and 35 °C for 16–20 h. The growth in the four plates is checked and interpreted for whether or not there is growth, so that it is intermediate if it grows in the plate with 2 μg/mL, or resistant if it grows in the plate with 4 μg/mL [34].

## 5. Conclusions

The genus *Aeromonas* is a bacterium widely distributed in the environment. Its presence in various ecosystems and ability to cause infections in animals and humans makes it a bacterium of interest in the study related to the appearance of strains resistant to antimicrobials. The presence of antimicrobial-resistant isolates from the first line of care, and those of the last alternative, such as colistin, detected in the clinical area, in animals or in the environment, demonstrates that the resistance comes from the exposure of bacteria to antimicrobials in clinical care, but it is also suggested that resistance originates in the environment. In addition, it is important to study *Aeromonas* isolates with colistin resistance from different sources, and also with the detection of the *mcr* genes.

## Figures and Tables

**Table 1:** Colistin activity spectrum on Gram-negative bacteria.

Sensitive	Resistance	Variables
Escherichia coli * Klebsiella pneumoniae * Pseudomonas aeruginosa * Acinetobacter baumannii * Salmonella spp. *Shigella* spp. *Legionella pneumophila* *Haemophilus influenzae* *Bordetella pertussis* *Prevotella* spp.^a^ *Fusobacterium* spp.^a^	*Proteus* spp. *Burkholderia* spp. *Helicobacter pylori* *Neisseria meningitidis* *Bacteroides fragilis* ^a^	*Stenotrophomonas maltophilia* *Moraxella catarrhalis* *Vibrio* spp. *Aeromonas* spp.

* Sensitive Gram-negative bacilli with reported colistin resistance. ^a^ Strict anaerobic bacteria. Modified from the literature [6].
